# Real-World Outcomes of Upfront Abiraterone in Metastatic Castration-Sensitive Prostate Cancer Patients at a Tertiary Care Hospital

**DOI:** 10.7759/cureus.86518

**Published:** 2025-06-22

**Authors:** Muhammad Awais Majeed, Ayesha Alam, Maryam Imran, Shakeel Muzaffar, Muhammad Ahsan Jamil, Noor Ul Ain, Muhammad Usman Shabbir, Umer Salman, Sameen Bin Naeem

**Affiliations:** 1 Oncology, Shaukat Khanum Memorial Cancer Hospital and Research Centre, Lahore, PAK; 2 Physiology, Al-Aleem Medical College, Lahore, PAK; 3 Medical Oncology, Shaukat Khanum Memorial Cancer Hospital and Research Centre, Lahore, PAK; 4 Internal Medicine, Shaukat Khanum Memorial Cancer Hospital and Research Centre, Lahore, PAK

**Keywords:** abiraterone acetate, castration-resistant, castration-sensitive, overall survival, progression-free survival

## Abstract

Background

Metastatic castration-sensitive prostate cancer is defined as prostate cancer with de novo metastatic disease that responds to androgen deprivation therapy by keeping the testosterone levels low. Endogenous androgen synthesis is further blocked by abiraterone acetate along with prednisolone and indicated in patients with metastatic castration-sensitive prostate cancer. However, over time, these patients will become castration-resistant. The time from castration-sensitive to castration-resistant in our population is short, which calls for further investigation on a larger scale to explore factors such as genetics and environmental influences that may play a significant role.

Methodology

This retrospective study involved 47 adult patients aged 40 years and older. It focused exclusively on patients who were presented with de novo metastatic castration-sensitive disease and were treated with upfront abiraterone acetate. The study was carried out at the Department of Medical Oncology, Shaukat Khanum Memorial Cancer Hospital and Research Centre, Lahore, Pakistan. Patient data spanning 10 years, from 2014 to 2024, was collected from hospital records.

Results

The cohort demonstrated a median progression-free survival (PFS) of 20.7 months and a median overall survival (OS) of 38.4 months. These outcomes represent the entire study population, irrespective of subgroup classification. Different subgroup analyses do not show any statistically significant difference.

Conclusion

In our study, OS and PFS were lower than those reported in landmark studies conducted on similar populations in Western countries. This disparity highlights the need for further research in subcontinental populations to investigate potential contributing factors, including environmental influences or genetic variations.

## Introduction

Prostate cancer is a heterogeneous disease with incidence rates that vary significantly across the world, from 6.3 to 83.4 per 100,000 people, and is the third most diagnosed malignancy [1]. In the United States, approximately 3% of all new prostate cancer cases are metastatic castration-sensitive cancers [2]. Historically, androgen deprivation therapy has been the standard of care for the treatment of prostate cancer patients. It includes bilateral orchiectomy or luteinizing hormone-releasing hormone analogues, with or without first-generation androgen receptor inhibitors [3]. Although many patients initially respond to androgen deprivation therapy, the majority of patients with metastatic disease progress to castration-resistant prostate cancer within a median of approximately one year [4-6]. This resistance is primarily driven by continued adrenal androgen production, which reactivates androgen receptor signaling and promotes increased intertumoral testosterone synthesis. Other contributing mechanisms involve changes in the structure and function of androgen receptors and the engagement of parallel steroidogenic pathways [3]. 

Cytochrome P450c17 plays a key role in androgen biosynthesis and is inhibited by abiraterone acetate, a prodrug converted to abiraterone. Emerging research suggests that its active metabolite, D4A, contributes to antitumor activity by inhibiting several steroidogenic enzymes and acting as an androgen receptor antagonist [7,8]. Metastatic castration-resistant prostate cancer patients have been shown to receive significantly increased overall survival (OS) and additional clinical benefits with abiraterone acetate (hereafter called abiraterone) in combination with prednisone [9]. Based on these results, the role of abiraterone was studied in de novo metastatic castration-sensitive patients, which showed prolonged OS and longer radiographic progression-free survival (PFS) [10]. We analyzed how treatment with abiraterone influenced OS and PFS among our patients.

## Materials and methods

Study design and participants 

This retrospective study aimed to assess PFS and OS, as well as the relationship between patient and disease factors and these outcomes in metastatic castration-sensitive prostate cancer (mCSPC) patients treated with abiraterone. The Institutional Review Board of Shaukat Khanum Memorial Cancer Hospital and Research Centre (SKMCH&RC), Lahore, Pakistan (approval number: EX-06-02-25-01), approved this as a retrospective study and granted a waiver of informed consent. For the collection of data, the hospital information system of our institute was accessed, using a keyword search for abiraterone. A total of 47 patients fulfilled the study's inclusion criteria.

Eligible patients were those with de novo metastatic castration-sensitive disease, diagnosed by histopathology and prostate-specific antigen (PSA) levels, and treated with abiraterone therapy at SKMCH&RC between 2014 and 2024. All patients who fulfilled the inclusion criteria were included in the study.

Methods 

Patients' medical records present in the electronic hospital information system were accessed to obtain data on patients' demographics and disease characteristics. Electronic reports and physician notes were reviewed to confirm histopathology, disease stage, date of initiation of abiraterone therapy, duration of treatment, date of progression, date of last follow-up, and date of death.

We defined PFS and OS as the primary outcomes of our study and subgroup analysis of the effect of abiraterone on various patient and disease factors on PFS and OS as secondary outcomes. 

OS was assessed based on the interval duration between the date of starting abiraterone and the date of last follow-up and patient status at the last follow-up (alive or dead). Patients who were alive till the end of data collection (December 1, 2024) were considered censored in the survival analysis.

PFS was assessed by the interval between the dates of the start and end of abiraterone in cases of patients with progressive disease at the end of therapy. Those patients who had no evidence of progression were censored in PFS till the date of last follow-up (December 1, 2024). The PFS was defined as either an increase in PSA levels or evidence of radiological disease progression.

Statistical analysis 

Data was compiled and analyzed using IBM SPSS Statistics for Windows, Version 26.0 (Released 2019; IBM Corp., Armonk, New York, United States). Survival analysis was performed using the Kaplan-Meier method, with p-values calculated by the log-rank test; a p-value less than 0.05 was considered statistically significant.

## Results

The study included 47 patients. Among them, 14 patients (29.8%) had low-volume disease, while 33 patients (70.2%) presented with high-volume disease. Visceral metastases were observed in 13 patients (27.7%), and 21 patients (44.7%) had bone-only disease. Nodal metastases were present in 33 patients (70.2%). Regarding Gleason scores, 12 patients (25.5%) had a score less than 8, while 35 patients (74.5%) had a score of 8 or above. Based on PSA levels, 30 patients (63.8%) had values less than 100 ng/mL, whereas 17 patients (36.2%) had PSA levels equal to or greater than 100 ng/mL. This is shown below in Table 1.

**Table 1:** Clinical characteristics of patients PSA: prostate-specific antigen

Category	Subcategory	Patients (n, %)
Total patients	-	47 (100%)
Disease volume	Low-volume	14 (29.8%)
	High-volume	33 (70.2%)
Metastasis type	Visceral metastases	13 (27.7%)
	Bone-only disease	21 (44.7%)
	Nodal metastases	33 (70.2%)
Gleason score	<8	12 (25.5%)
	≥8	35 (74.5%)
PSA level	<100 ng/mL	30 (63.8%)
	≥100 ng/mL	17 (36.2%)

Primary outcomes

PFS

**Figure 1:**
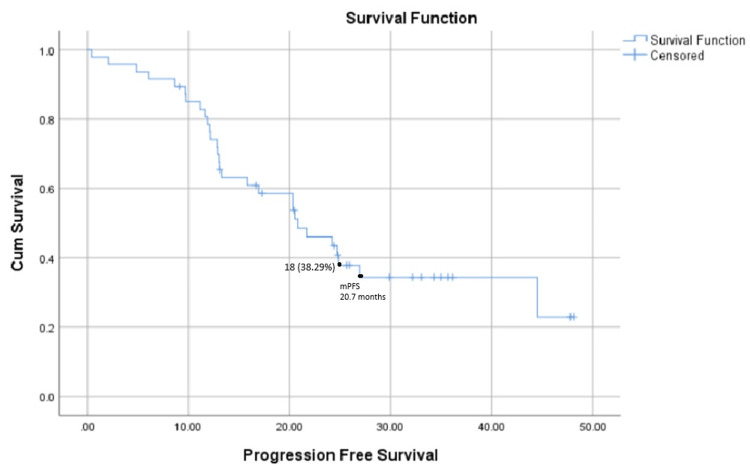
Progression-free survival in months from the commencement of abiraterone

The median PFS for the group was 20.7 months, and 18 patients (38.29%) remained progression-free at the time of the last follow-up. This is shown below in Figure 1.

OS

**Figure 2:**
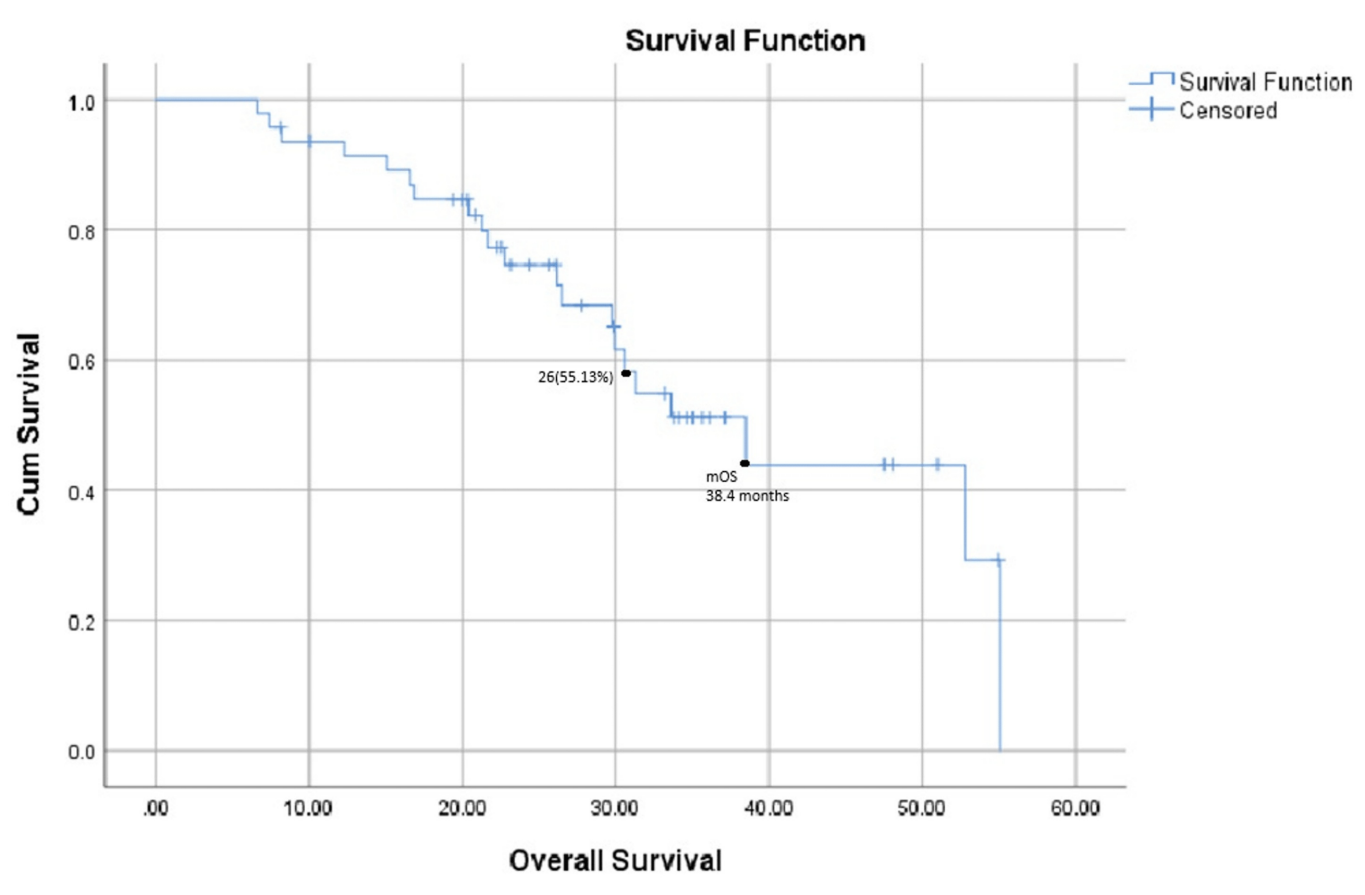
Overall survival in months from the commencement of therapy to the date of death or last follow-up

The median OS for the entire study population was 38.4 months, with 26 patients (55.13%) still alive at the last follow-up. This is shown below in Figure 2.

Secondary outcomes

Difference of PFS Based on Subgroup Analysis 

**Figure 3:**
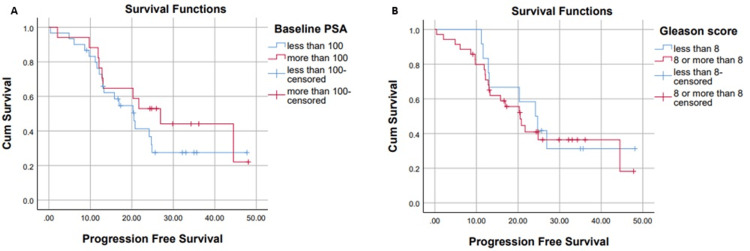
Progression-free survival on the basis of PSA levels (A) and Gleason score (B) PSA: prostate-specific antigen

In the subgroup analysis, patients with a Gleason score ≤7 had a median PFS of 24.1 months, compared to 20.5 months in those with a Gleason score >7 (p=0.7). The median PFS for patients with a PSA level <100 ng/mL was 20.5 months, while those with PSA ≥100 ng/mL had a median PFS of 26.9 months (p=0.3). This is shown below in Figure 3.

**Figure 4:**
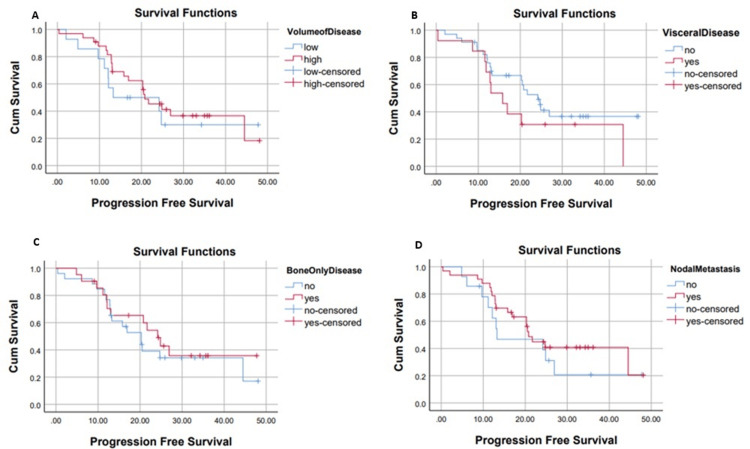
Progression-free survival in months from the commencement of abiraterone with regard to volume of disease (A), visceral disease (B), bone-only disease (C), and nodal metastasis disease (D)

Patients with low-volume disease had a median PFS of 13.27 months, whereas those with high-volume disease demonstrated a median PFS of 20.7 months (p=0.53). Patients with visceral metastases had a shorter median PFS of 15.8 months, compared to 24.1 months in those without visceral involvement (p=0.1). Among patients with bone-only disease, the median PFS was 20.3 months, whereas those with both bone and other metastatic sites had a median PFS of 24.1 months (p=0.52). Similarly, patients with nodal metastasis had a median PFS of 20.7 months, while those with no nodal metastasis had a median PFS of 13.2 months (p=0.3). This is shown below in Figure 4.

Differences in OS Based on Subgroup Analysis

**Figure 5:**
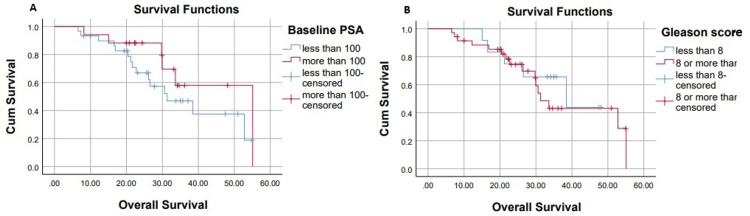
Overall survival on the basis of PSA levels (A) and Gleason score (B) PSA: prostate-specific antigen

In the subgroup analysis for OS, patients with a Gleason score <8 demonstrated a median OS of 38.4 months, compared to 31.3 months in those with a Gleason score ≥8 (p=0.5). The median OS for patients with PSA levels <100 ng/mL was 31.3 months, whereas those with PSA ≥100 ng/mL had a longer median OS of 55 months (p=0.1). This is shown below in Figure 5.

**Figure 6:**
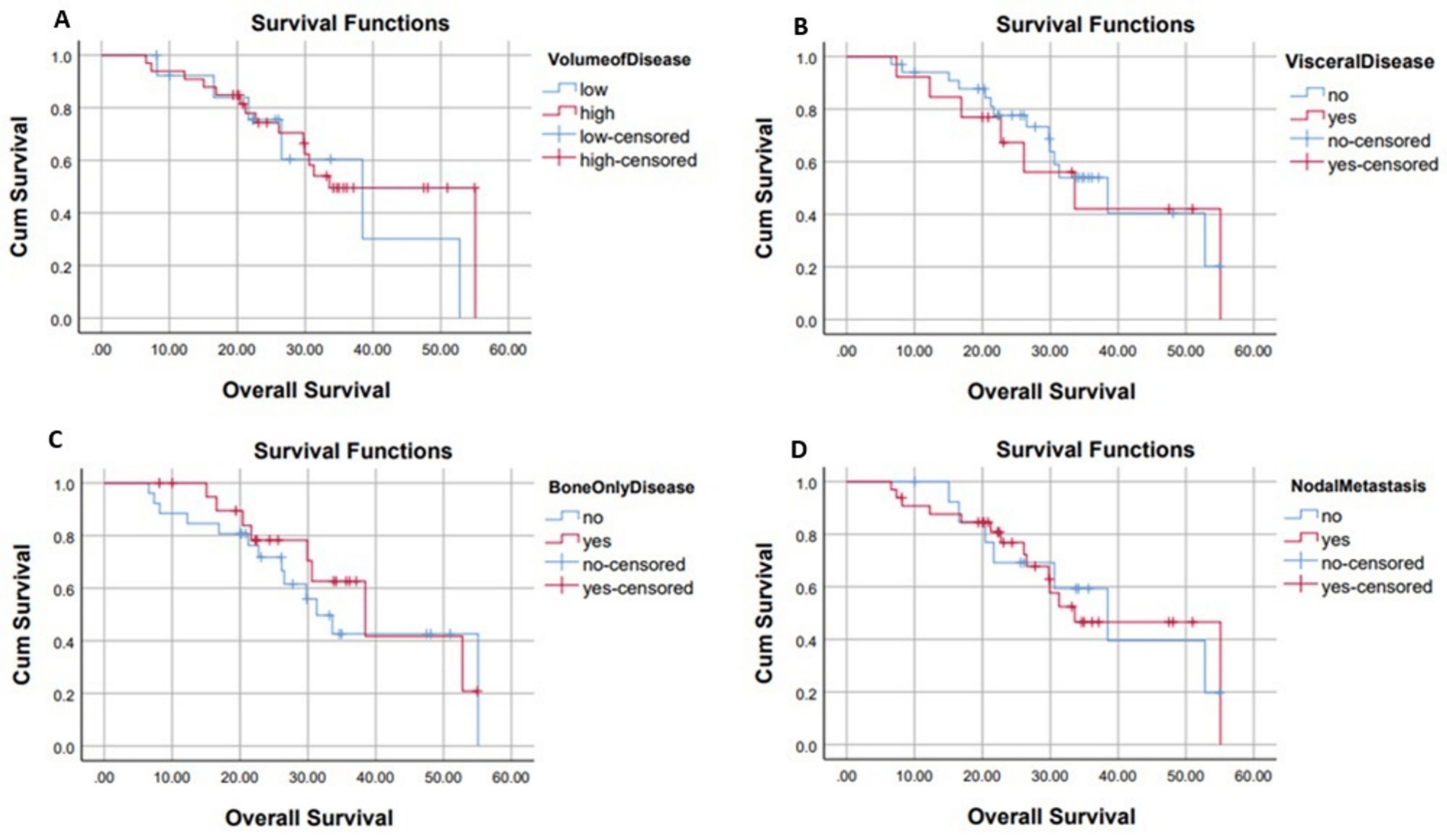
Overall survival in months from the commencement of abiraterone with regard to volume of disease (A), visceral disease (B), bone-only disease (C), and nodal metastasis disease (D)

Patients with low-volume disease had a median OS of 38.4 months, while those with high-volume disease had a median OS of 33.6 months (p=0.5). Patients with visceral metastases had a median OS of 33.6 months, while those without visceral involvement had a median OS of 38.4 months (p=0.8). Among patients with bone-only disease, the median OS was 38.4 months, compared to 31.3 months in those with both bone and other metastatic sites (p=0.5). Similarly, patients with nodal metastasis had a median OS of 33.6 months, while those with no nodal metastasis had a median OS of 38.4 months (p=0.9). This is shown below in Figure 6.

The difference in median PFS and median OS in subgroup analysis is as shown in Table 2.

**Table 2:** Subgroup analysis of median PFS and median OS PSA: prostate-specific antigen; PFS: progression-free survival; OS: overall survival

Subgroup	Category	Median PFS (months)	P-value (PFS)	Median OS (months)	P-value (OS)
Disease volume	Low-volume	13.27	0.53	38.4	0.5
	High-volume	20.7		33.6	
Gleason score	≤7	24.1	0.7	38.4	0.5
	>8	20.5		31.3	
PSA level (ng/mL)	<100	20.5	0.3	31.3	0.1
	≥100	26.9		55.0	
Visceral disease presence	Present	15.8	0.1	33.6	0.8
	Absent	24.1		38.4	
Metastatic sites	Bone-only	20.3	0.52	38.4	0.5
	Bone+other sites	24.1		31.3	

## Discussion

This study evaluates PFS and OS in a cohort of 47 patients with mCSPC treated with abiraterone and analyzes outcomes across clinical and pathological subgroups. The cohort demonstrated a median PFS of 20.7 months and a median OS of 38.4 months. These results are comparable to outcomes reported in previous real-world studies and clinical trials [11]. In a comparable cohort, a median PFS of 23 months with abiraterone treatment was observed, corroborating our results [12].

In contrast, larger phase 3 trials have shown more favorable outcomes. The LATITUDE trial reported a median PFS of 33 months and a median OS of 53.3 months among newly diagnosed high-risk mCSPC patients [13]. Similarly, the STAMPEDE trial reported a median OS of 76.6 months and a median PFS of 42 months in the abiraterone-treated group [14]. The relatively shorter PFS and OS observed in our cohort may be attributable to a smaller sample size, genetic variability within the population, and more advanced or aggressive disease characteristics at baseline. 

Despite most of our patients presenting with high-volume disease (70.2%), subgroup analysis showed no statistically significant differences in PFS (20.7 vs. 13.3 months; p=0.53) or OS (33.6 vs. 38.4 months; p=0.5) between high- and low-volume groups. This contrasts with findings from larger studies, such as STAMPEDE, where high-volume disease was associated with a worse prognosis [15]. The lack of statistical significance in our analysis may reflect the limited sample size and variability in baseline characteristics. 

A notable trend was observed in Gleason scores. Patients with Gleason scores ≤7 had a longer median PFS (24.6 months) and OS (38.4 months) compared to those with scores ≥8 (PFS: 20.5 months; OS: 31.3 months), although these differences were not statistically significant (p=0.1 and p=0.5, respectively). These findings differ from those of Wu et al., who reported that higher Gleason scores were associated with a more favorable response to androgen receptor-targeted therapies [16]. 

Baseline PSA levels did not significantly impact outcomes. Patients with PSA ≥100 ng/mL had a longer median PFS (26.9 months) and OS (55 months) compared to those with PSA <100 ng/mL (PFS: 20.5 months; OS: 31.3 months; p=0.1). These findings differ from the STAMPEDE trial, which reported that elevated PSA levels were associated with worse survival outcomes [6]. This difference may be attributed to the different PSA stratification methods used in our study, as well as the smaller sample size.

The presence of visceral metastases, while not statistically significant (p=0.1), was associated with a shorter median PFS (15.8 months) compared to patients without visceral involvement (24.1 months). This trend supports other findings suggesting visceral disease may indicate more aggressive tumor biology, even with abiraterone treatment [17]. However, Mittal et al. reported improved PFS and OS in patients with visceral metastases treated with a triple-therapy approach including abiraterone, suggesting the potential for therapeutic intensification to overcome this poor prognostic feature [18]. 

Patients with bone-only metastases had a median PFS of 20.3 months, which was not significantly different from the 24.1 months observed in those with both bone and other metastases (p=0.1). This contrasts with the findings by Urabe et al., who reported better outcomes in mCSPC patients with bone-only disease [19]. 

While our results are generally aligned with real-world evidence and selected trial data, the study's retrospective nature and limited sample size restrict the generalizability of its conclusions. Further prospective, multicenter studies with larger cohorts are warranted to validate these results and refine patient selection for abiraterone-based treatment in mCSPC in resource-limited settings where cost-effective therapeutic decisions are crucial. 

## Conclusions

The study concludes that the real-world outcomes of upfront abiraterone in mCSPC are lower within our population than those observed in clinical trials. Additionally, there was no statistically significant difference between subgroups in our study. To confirm these findings and better understand which patient populations may derive the most benefit from this treatment, future research with larger, preferably prospective, cohorts is necessary.
